# Correlates of fasting blood glucose among children living with hiv in a Nigerian tertiary hospital: a cross-sectional study

**DOI:** 10.1186/s12887-020-02335-y

**Published:** 2020-10-02

**Authors:** Ijeoma Onyinye Ohuche, Ugo Nnenna Chikani, Elizabeth Eberechi Oyenusi, Justus Uchenna Onu, Abiola Oduwole

**Affiliations:** 1 Department of Paediatrics, Niger Foundation Hospital, Enugu, Nigeria; 2 Department of Paediatrics, University of Nigeria Teaching Hospital, Enugu, Nigeria; 3 Paediatric Endocrinology Training Centre for West Africa, LUTH, Lagos, Nigeria; 4 Department of Paediatrics, Lagos University Teaching Hospital, Lagos, Nigeria; 5 Department of Paediatrics, College of Medicine, University of Lagos, Lagos, Nigeria; 6 Mental Health Unit, Department of Internal Medicine, Nnamdi Azikiwe University, Nnewi, Anambra State Nigeria

**Keywords:** Fasting, blood glucose, HIV, Children

## Abstract

**Background:**

There is growing concern as regards the emergence of metabolic disorders among children living with the Human Immunodeficiency Virus (HIV) worldwide. However, there is paucity of data on the correlates of metabolic indices among HIV-positive children in Africa.

**Methods:**

This study examined 84 HIV-positive children on HAART recruited from the paediatric infectious diseases clinic of the University of Nigeria Teaching Hospital for blood glucose levels using finger-prick testing with an Accu-check glucose meter and test strips. Clinical information was obtained via clinical history and medical records. Data was analyzed to examine the relationship between FBG and the classes of HAART, duration of illness and treatment using analysis of variance (ANOVA).

**Results:**

FBG was significantly associated with the classes of HAART (_x_^2^=12.4,* p* = 0.017). In addition, there was a significant association between FBG and duration of illness [F(2, 81) = 6.0; *P *= 0.004], as well as FBG and duration on HAART [F(2, 81) = 7.9; *P *= 0.001]. However, duration on HAART and type of HAART were the significant predictors of FBG in this study accounting for 10.5% and 4.1% of the variance, respectively.

**Conclusions:**

There is a greater risk of dysglycemia in paediatric patients with a longer cumulative exposure to HAART. Routine blood glucose checks among children on HAART, especially those who have received HAART for a longer duration of time may therefore be useful in their management.

## Background

Nigeria has the second largest HIV pandemic burden worldwide, [1] and in 2017, accounted for half of all children and adolescents living with HIV in West and Central Africa [1]. These children are now more likely to survive and have longer lifespans, as a consequence of improved awareness and screening, as well as success with the use of highly-active anti-retroviral therapy (HAART). However, increased survival of these patients has its implications, as this has been associated with a concurrent emergence of metabolic disorders over time [2–4]. These metabolic aberrations include disorders in glucose and lipid metabolism, as well as lactic acidosis, and have been largely associated with the use of HAART [4]. These metabolic disturbances carry with them, a series of risk factors for cardiovascular disease, such as central obesity, atherogenic dyslipidemia, hypertension, and impaired insulin and glucose metabolism with the risk of cardiovascular-related mortality and morbidity.

Disorders of glucose metabolism are especially important among the cardio-metabolic consequences of HAART, since pre-diabetes (comprising impaired fasting glucose and impaired glucose tolerance) has been identified as a well-known risk factor for the development of diabetes and cardiovascular disease, which would lead to increased morbidity and mortality among children with HIV, if present [5, 6]. Indeed, development of pre-diabetes is regarded as the central event that is significantly associated with subsequent development of other cardiometabolic problems such as hypertension and dyslipidemia [5]. Early identification of prediabetes and its risk factors, especially among predisposed populations such as the study population, is therefore of paramount importance, and represents an effective preventive measure for the development of cardiometabolic complications over time in this patients.

Even though there is modest literature regarding the occurrence of metabolic disturbances in HIV-infected children, [2–4, 7] the mechanism underlying the increased prevalence of these abnormalities in children with HIV-infections is nonetheless, still unclear. Furthermore, very few studies have addressed the issue of glucose abnormalities among children living with HIV in developing countries, such as Nigeria. There is therefore a need to explore the relationship between HAART and the development of glucose abnormalities in the paediatric population living with HIV in our locality. Since glucose abnormalities increase the risk of development of type 2 diabetes and macrovascular disease in predisposed individuals, the need for such data cannot be over-emphasized. The knowledge obtained from this study will enrich our knowledge of HIV management and its long-term consequences, and will therefore improve management outcomes for children living with HIV in the long run.

Children living with HIV in our facility are placed on two broad categories of treatment: (1) A non-nucleoside- reverse transcriptor-inhibitor (NNRTI) - based regimen, consisting of a non-nucleoside reverse transcriptor inhibitor and two nucleoside reverse transcriptor inhibitors; or (2) a protease-inhibitor –based regimen, consisting a boosted protease inhibitor and two nucleoside reverse transcriptor inhibitors. We therefore set out to determine the relationship between these two broad categories of HAART regimen (PI-based or NNRTI-based), duration on HAART and duration of illness; and the fasting blood glucose levels of HIV-positive children receiving treatment in our facility.

## Methods

This was a cross-sectional study of 84 HIV-positive children aged 5 to 18 years, receiving HAART. They were recruited from the Paediatric Infectious Diseases Clinic of the University of Nigeria Teaching Hospital, Enugu from January, 2018 to April, 2018. According to statistics from the records department from 2015 to 2017, about 31 children living with HIV are seen in the clinic every month. This translates to about 124 cases in 4 months.

Consecutive clinic attendees who met the study eligibility criteria were included. Participants were included if they were HIV-positive, aged between 5 and 18 years and had received HAART for at least one year. Children with known diabetes mellitus prior to commencement of HAART and those on chronic steroid therapy were excluded from the study. Newly-diagnosed patients were also excluded from the study.

A total of 122 participants were approached for the study. However, after further evaluation, 17 were excluded because they did not give consent. An additional 21 were excluded because they were newly-diagnosed; leaving a total of 84 participants that completed the study.

Caregivers of recruited subjects gave written consent for the study after a full explanation of the details of the study, and children seven years of age and above gave assent in addition. Caregivers were given an interviewer-administered questionnaire to fill, which provided information regarding the subject’s age, gender, and family history of diabetes. Other clinical details of the subjects such as: age at diagnosis of HIV, current treatment regimen, past treatment regimen and duration of HAART, were obtained from the subjects’ case notes.

In computing the required sample size for the study, we used the findings from Santiprabhob et al. [8] for the following reasons: Firstly, they studied a similar population using the same design. Secondly, both studies used institution-based patients (care-seekers) as against a community sample. Thirdly, both studies were carried in low and middle income (LAMI) countries. Based on their finding that the prevalence of pre-diabetes among patients with HIV was 22.1% and level of precision set at 5%, we computed the required minimum sample size by using this figure to substitute in the Araoye formula for proportion in a single group (z^2^pq/d^2^) and arrived at 265. However, the expected proportion of children with HIV-infection on HAART attending the clinic in the study site over the 4 month period of the study was 124. We further substituted into a final sample size formula for a finite population (nf = n/1 + n/N) and arrived at 80 as the minimum sample size.

### Blood glucose testing

Blood glucose measurements are ordinarily not carried out for our patients at their routine clinic visits. An appointment for the measurement of blood glucose was therefore fixed on a day convenient for both the subject and the caregiver. They were given instructions for patient to fast on the morning of the test. The last meal was eaten between eight and ten hours prior to the blood test. Measurement of fasting blood glucose (FBG) was done using finger prick testing with an Accu-check glucometer and glucose test strips. The procedure was first explained to the subject, and then the subject’s finger was gently pricked with a single-use Accu check Safe-T Pro Uno lancet. A drop of blood was then placed on the disposable test strip, and blood glucose levels were read off the glucose meter in mg/dl. The test result was then communicated to the subject and the caregiver. Bleeding was controlled by the application of gentle pressure using a dry cotton swab.

Following the 2014 ISPAD consensus guidelines, [9] a FBG value less than 100 mg per deciliter was documented as normal, while impaired FBG was defined as a value between 100 and 125 mg per deciliter. Diabetes was defined as a FBG level of 126 mg per deciliter or higher. Normal blood glucose values were further subdivided into high normal (˃ 87 mg/dl to 99 mg/dl) and moderate normal (87 mg/dl and below) values, the former having been associated with the risk of pre-diabetes [10–12]. Children with impaired fasting glucose were referred to the Paediatric Endocrinology Clinic of the Hospital for further evaluation.

### Ethical Considerations

The study was approved by the Health Research Ethics Committee of the University of Nigeria Teaching Hospital, Enugu.

### Statistical Analysis

Statistical analysis was carried out with the Statistical Package for Social Sciences (IBM-SPSS), version 20. First, data was cleaned and the normality of distribution was checked using Shapiro Wilk test. The relationship between FBG (categorized), type of HAART, family history of diabetes (positive/negative), and gender (male/female) was determined using Fisher’s Exact 2-sided test, while the relationship between FBG and age, duration of illness and treatment were determined using analysis of variance (ANOVA) with Bonferroni Post-Hoc multiple comparisons. Multiple stepwise regression analysis was used to determine the socio-demographic and clinical predictors of fasting blood glucose.

The choice of multiple linear regression (i.e., handling fasting blood glucose as a continuous variable) instead of multinomial logistic regression (i.e., handling as a categorical variable) was due to the small number of participants in the category of impaired glucose tolerance.

## Results

Table 1 shows the socio-demographic and clinical characteristics of the 84 participants. The mean age of the participants was 12.4 ± 3.5 years, with an equal male and female ratio. The mean duration of the illness and duration of HAART treatment were 6.9 ± 3.3 and 6.5 ± 3.2 years, respectively. Majority (91.7%) of the participants were on an NNRTI-based HAART regimen. The mean FBG among the study participants was 83.3 mg/dl. There was a low prevalence of impaired fasting glucose among the study population (3.6%). Though female subjects had a slightly higher mean FBG than the males, both genders did not differ significantly with respect with their fasting blood glucose level (*p* = 0.16).

The association between HAART regimen, family history of diabetes, gender and fasting blood glucose is shown in Table 2. The table shows there was a significant relationship between the fasting blood glucose and the class of HAART regimen (*p* = 0.017). However, there was no significant association between family history of diabetes, gender and the glycemic profile of the participants.

**Table 1:** Socio-demographic and clinical characteristics of the participants

**Variables**	**n(%)**	**Mean(S.D)**	**95% CI**
**Mean Age (years)**		12.4(3.5)	11.7-13.1
**Age Group**			
Pre-adolescence	20(23.8)		
Adolescence	64(76.2)		
**Gender **			
Male	42(50.0)		
Female	42(50.0)		
**Family History of DM**			
Negative	74(88.1)		
Positive	10(11.9)		
Mean Age at Diagnosis		5.6(3.9)	4.8-6.4
Mean Duration of Illness (years)		6.9(3.3)	6.2-7.6
Age at Initiation of HAART		5.9(4.0)	5.1-6.7
Mean Duration on HAART		6.5(3.2)	5.8-7.2
**Fasting blood glucose (mg/dl)**		Overall= 88.3 (9.9)	86.2-90.4
		Males = 86.8 (8.2)	85.8-90.8
		Females = 89.9 (11.2)	86.5-93.3
		PA= 85.0 (2.2)	80.7-89.3
		Adolesc = 89.4 (1.2)	87.0-91.8
High Normal	55 (65.5)		
Moderate Normal	26 (31.0)		
Impaired Fasting Glucose	3(3.6)		
**Type of HAART Regimen**			
PI-based	8 (9.5)		
NNRTI-based	76 (90.5)		

*NB: HAART *Highly-active anti-retroviral therapy*, NNRTI *Non-nucleoside reverse transcriptase inhibitors*, PI *Protease inhibitors*, *gender vs. FBG (*p*=0.17),Age category vs. FBG (*p*=0.08), *DM *Diabetes Mellitus, *PA *Pre-adolescence, *Adolesc *Adolescence

**Table 2:** Relationship between family history of diabetes, gender, type of HAART and glycemic profile of the participants

**Variables **	**Fasting Blood Glucose**			**Statistics **
	Moderate Normal	High Normal	Impaired Fasting Glucose	
**FHD**				
Positive	2(7.7%)	8(14.5%)	0(0.0%)	*p*=0.652*
Negative	24(92.3%)	47(85.5%)	3(100.0%)	
**Total**	26(100.0%)	55(100.0%)	3(100.0%)	
**Gender **				
Male	15(57.7%)	27(49.1%)	0(0.0%)	*p*=0.190*
Female	11(42.3%)	28(50.9%)	3(100.0%)	
**Total **	26(100.0%)	55(100.0%)	3(100.0%)	
**Type of HAART**				
NNRTI-based	25(96.2%)	50(90.9%)	1(33.3%)	*p*=0.017*
PI-based	1(3.8%)	5(9.1%)	2(66.7%)	
**Total **	26(100.0%)	55(100.0%)	3(100.0%)	

*FHD *Family History of Diabetes; *= Fisher’s Exact (2-sided) test, HAART Highly-Active Anti-Retroviral Therapy*, NNRTI *Non-Nucleoside Reverse Transcriptase Inhibitors*,*and *PI *Protease Inhibitors

The relationship between fasting blood glucose and clinical variables such as duration of illness, age at diagnosis, age at onset of treatment, duration on current treatment and total duration on HAART is shown in Table 3. The mean of duration of illness for participants’ with moderate high, high normal and impaired fasting blood glucose were 5.19 ± 0.46 (95% CI, 4.24–6.15), 7.56 ± 0.44 (95% CI, 6.67–8.45) and 9.00 ± 2.52 (95% CI, -1.83-19.83) years, respectively [F(2, 81) = 6.0; *P* = 0.004). Bonferroni Post-Hoc multiple comparisons show that the significance was between high normal and moderate normal fasting blood glucose (*p *= 0.005). Also, as shown in Table 3, the mean (SD) duration on HAART for participants’ with impaired, high normal and moderate normal fasting blood glucose were 9.33 ± 2.03 (95% CI, 1.61–18.06), 7.17 ± 0.43 (95% CI, 6.31–8.03), and 4.65 ± 0.45 (95% CI, 373 − 5.57) years, respectively [F(2, 81) = 7.9; *P* = 0.001). Bonferroni Post-Hoc multiple comparisons show that the significance was between high normal and moderate normal fasting blood glucose (*p* = 0.002) and moderate and impaired fasting blood glucose (*p* = 0.03).

**Table 3:** Relationship between fasting blood glucose, age at diagnosis, age at treatment, duration of current treatment and total duration on HAART

**Variables **	**Fasting Blood Glucose**			**F-stat**	***p-value***
	Moderate Normal	High Normal	Impaired Fasting Glucose		
**Age at diagnosis (years)**				1.62	0.204
Mean (SD)	6.42(0.74)	5.31(0.53)	2.66(0.33)		
95%CI	4.88-7.97	4.24-6.37	1.23-4.10		
**Duration of illness (years)**				6.01	0.004
Mean (SD)	5.19(0.46)	7.56(0.44)	9.00(2.52)		
95%CI	4.24-6.15	6.67-8.45	-1.83-4-19.83		
**AAT (years)**				1.93	0.152
Mean (SD)	4.50(0.46)	6.75(0.44)	8.67(0.34)		
95%CI	3.55-5.45	5.87-7.63	-0.74-18.07		
**DOH (years)**				7.94	0.001
Mean (SD)	4.65(0.45)	7.17(0.43)	9.33(2.03)		
95%CI	3.73-5.57	6.31-8.03	1.61-18.06		
**Age **				1.98	0.294
Mean (SD)	11.62(0.71)	12.87(0.45)	11.67(2.19)		
95%CI	10.15-13.08	11.96-13.78	2.26-21.07		

*SD *Standard Deviation*, CI *Confidence Interval, *AAT *Age at Onset of Treatment; and*, DOH *Duration on HAART

The socio-demographic and clinical factors were tested as predictors of fasting blood glucose among children with HIV using stepwise multiple regression analysis, because of the need to enter separately the clinical and socio-demographic variables. First, the regression model was a good fit for the data [F (1, 82) = 940.87, *p* = 0.002]. The result showed that of the two socio-demographic variables (age and gender) and 5 clinical variables (i.e., age at diagnosis, duration of illness, duration on HAART, family history of diabetes, and type of HAART), duration on HAART and type of HAART entered the regression equation. They accounted for 10.5% of the variance in the fasting blood glucose (standardized β-coefficient = 0.323, *p* < 0.001, R^2^ = 0.105) and 4.1% (standardized β-coefficient = 0.229, *p* < 0.001, R^2^ = 0.041), respectively, as shown in Table 4.

**Table 4:** Summary of stepwise regression result of socio-demographic and clinical predictors of fasting blood glucose

Dependent variable	Significant predictors	Standardized β coefficient	t-stat	*p*-value	Variance (R^2^%)
Fasting Blood Glucose (mg/dl)	Duration on HAART	0.341	3.28	<0.001	10.5
	Type of HAART	0.229	2.128	<0.001	4.1
	F-stat. = 940.87; df = 1, 82			Prob (F-stat.) = 0.002	

R2 (%)= Coefficient of multiple determination

## Discussion

This study was aimed at determining the relationship between the fasting blood glucose of the subjects and their indices of illness and management such as: the type of HAART regimen being received, duration of HIV infection, duration on HAART therapy and family history of diabetes.

The highlights of the findings of this cross-sectional study of Nigerian children living with HIV and receiving HAART are as follows: (1) The prevalence of impaired fasting blood glucose was 3.6% (95% CI, 3.2–3.7%); (2) The relationship between fasting blood glucose and family history of diabetes mellitus was not significant; (3) The type of HAART regimen (PI-based or NNRTI-based), duration on HAART, and duration of illness showed a significant association with fasting blood glucose levels; however, (4) Duration on HAART and type of HAART were the significant predictors of fasting blood glucose levels on multiple regression, after controlling for confounders.

The prevalence of dysglycemia documented in this study is low, when compared with that documented by previous studies, with a range of 5.0 to 24.6% [13–15]. However, most of the studies cited were carried out among adult populations, as previously stated, which differs from the present study that was carried out among the paediatric age group. The lower prevalence of dysglycemia in this population may result from the fact that abnormalities of glucose metabolism are less common in children than adults, since age has been shown as a risk factor for the development of glucose metabolic abnormalities [9]. This is largely attributed to the impairment of pancreatic beta-cell function which occurs as part of the normal aging process [9]. It is noteworthy that majority (87.5%) of patients receiving protease inhibitors had high-normal blood glucose or impaired fasting glucose, while two out of the three subjects (66.7%) with impaired fasting glucose were receiving a PI-based regimen.

### Fasting blood glucose and HAART regimen

PIs are said to be the anti-retrovirals most commonly associated with glucose abnormalities, [16] and an advisory warning was issued by the FDA in 1997 regarding the development of hyperglycaemia in patients on treatment with PIs. [17] Many studies have therefore been carried out among HIV-positive patients on protease inhibitors, comparing their blood glucose profile to that of HAART-naïve patients. These have shown an independent association between use of protease inhibitors and hyperglycemia [18–21]. Other studies have implicated protease inhibitors in the development of glucose intolerance among patients on HAART [22–25]. Protease inhibitors lead to insulin resistance by decreasing pancreatic beta cell response, and interfering with glucose transportation across the cell membrane [4]. Few paediatric studies have compared blood glucose profiles of children on the different classes of antiretroviral therapy [26, 27]. In the index study, the blood-glucose profiles of children on a PI-based regimen and those on an NNRTI-based regimen were compared, and most children on a PI-based were noted to have significantly high blood glucose values. Vigano et al. [28] documented similar findings in their 4-year follow up of children on HAART. Dejkhamron et al. [29] also documented a high prevalence of insulin resistance and pre-diabetes among children on PI-based regimens. Santiprabhob et al. [8] documented a prevalence of 22.1% for impaired fasting blood glucose among adolescents on a PI-based regimen. These studies showed similarities with the index study, in terms of methodology. The association between blood glucose and treatment with a PI-based regimen in this study was significant on multiple regression after controlling for confounders. This strengthens the findings that PI-based regimen is implicated in impaired glucose tolerance.

### Fasting Blood Glucose, Duration on HAART and Duration of HIV infection

In the index study, duration on HAART emerged the only predictor of dysglycemia following multiple regression analysis. The finding of significantly increasing fasting blood glucose levels with increasing duration of therapy on HAART is not surprising, and is in keeping with literature, in which some studies have associated the development of diabetes and glucose intolerance among HIV-positive patients with longer cumulative exposure to certain ARVs. [7] This finding is similar to that reported by Saves et al., [21] who documented a higher prevalence of diabetes among HIV-infected patients on HAART at 20 months following commencement of HAART when compared with 12 months post-commencement. This outcome may be explained by a greater toxicity of the anti-retroviral medications when taken over a longer period of time, and is in keeping with the recent emergence of metabolic abnormalities among HIV-positive patients receiving ARV therapy for longer periods.

Likewise, the index study documented that fasting blood glucose levels were shown to increase with longer duration of HIV infection. This finding supports the theory that HIV infection on its own may also alter normal glucose metabolism among infected patients via mechanisms such as pancreatic beta cell dysfunction and HIV-related inflammation and pro-inflammatory cytokines [30, 31]. Therefore the risk of glucose intolerance may be increased in patients who have harbored the infection for a longer period of time. However, this association did not remain significant on multiple regression analysis suggesting that the effect of duration of HIV-infection on fasting blood glucose may mediated by another factor such as duration of treatment.

### Limitations

One of the limitations of this study is the small number of participants with impaired fasting glucose, thereby making it inadequate for comparison. However, the magnitude of this problem may have been attenuated by the use of regression analysis which is not easily influenced by sample size, to test predictors of fasting blood glucose among the study participants. The second limitation is the problem related to sampling technique. Although the non-probability sampling method utilized in the study is adequate for such a sample study population, it is not suitable for a formal drug trial. Another limitation of this study was the use of institution based samples (care seekers); although it saved cost and time, and is the predominant methodology in the literature, a community sample would have been more representative.

## Conclusions

In conclusion, this study suggests a significant risk of dysglycemia in children receiving a PI-based HAART regimen, children who have received HAART for a longer duration of time and children who have been HIV-positive for a longer duration of time. Of these factors however, duration on HAART and type of HAART were the significant predictors of dysglycemia among HIV-treated children. Regular blood glucose checks among children on HAART, especially those who have received it for a longer duration, may therefore be useful in their management especially in resource-poor countries which may not afford routine blood glucose measurements for all children living with HIV.

## Abbreviations

ANOVA: Analysis of Variance
ARV: Antiretroviral
CI: Confidence Interval
FBG: Fasting Blood Glucose
FDA: Food and Drug Administration
HAART: Highly Active Antiretroviral Therapy
HIV: Human Immunodeficiency Virus
IBM: International Business Machines
ISPAD: International Society for Paediatric and Adolescent Diabetes
NNRTI: Non-nucleoside Reverse Transcriptase Inhibitor
PI: Protease Inhibitor
SD: Standard Deviation
SPSS: Statistical Package for the Social Sciences

## References

[1] NACA. (2017) ‘National Strategic Framework on HIV and AIDS: 2017–2020.

[2] Insulin resistance, hyperglycemia and diabetes on antiretroviral therapy. Guide for HIV/AIDS clinical care. Section 6: Comorbidities, coinfections and complications. https://aidsetc.org/guide/insulin-resistance-hyperglycaemia.

[3] Kalra S, Kalra B, Agrawal N, Unnikrishnan AG (2011). Understanding diabetes in patients with HIV/AIS. Diabetol metab syndr.

[4] Spagnuolo MI, Liguoro I, Guarino A (2014). Metabolic disorders in HIV-infected children. J Metabolic Synd.

[5] Umpathi KK, Thavamani A, Al-Kindi S (2019). Pre-diabetes in children and adolescents in the United States: Prevalence estimates and comorbidities - a population analysis. J Pediatr Endocrinol Metab.

[6] Huang Y, Cai X, Mai W, Li M, Hu Y. Association between prediabetes and risk of cardiovascular disease and all cause mortality: systematic review and meta-analysis. BMJ. 2016; 355. 10.1136/bmj.i5953. 10.1136/bmj.i5953 PMC5121106 27881363

[7] Brown TT, Cole SR, Li X, Kingsley LA, Palella FJ (2005). Antiretroviral therapy and the prevalence and incidence of diabetes mellitus in the multicentre AIDS Cohort Study. Arch Intern Med.

[8] Santiprabhob J, Tanchaweng S, Maturapat S, Maleesatharn A, Lermankul W, Sricharoenchai S, et al. Metabolic disorders in HIV-infected adolescents receiving protease inhibitors. Biomed Research International. 2017:2017:7481597. 10.1155/2017/7481597 PMC5331476 28293638

[9] Vincento de T. Age-related impairment of pancreatic beta-cell function: Pathophysiological and cellular mechanisms. *Front Endocrinol* (Lausanne). 2014;5:138. 10.3389/fendo.2014.00138 PMC4153315 25232350

[10] Nguyen QC, Srinivasan SR (2010). Fasting plasma glucose levels within the normoglycemic range in childhood as a predictor of prediabetes and type 2 diabetes in adulthood. The Bogalusa heart study. Arch pedatr Adolesc Med.

[11] Janghorbani M, Amini M (2011). Normal fating plasma glucose and risk of prediabetes and type 2 diabetes: The Isfahan diabetes prevention study. Rev Diabet Stud.

[12] Wang G, Arguelles L, Liu R, Zhang S, Brickman WJ, Hong X (2011). Tracking blood glucose and predicting prediabetes in Chinese children and adolescents: A prospective twin study. PLoS One..

[13] Shankalala P, Choolwe J, Samuel B, Michael V, Patrick K, Charles M (2017). Risk factors for impaired glucose or diabetes among HIV infected patients on ART in the Copperbelt Province of Zambia. J Diabetes Metab Disord.

[14] Duncan AD, Goff LM, Peters BS (2018). Type 2 diabetes prevalence and its risk factors in HIV: A cross-sectional study. PLoS ONE.

[15] Solomon MA, Assefa G, Solomon F, Mulugeta B, Abayneh GD, Nebiyu M (2016). Diabetes mellitus among HIV-infected individuals in follow-up care at university of Gondar Hospital, Northwest Ethiopia. BMJ Open.

[16] Rao MN, Lee GA, Grunfeld C (2006). Metabolic abnormalities associated with the use of protease inhibitors and non-nucleoside reverse transcriptase inhibitors. Am J Infect Dis.

[17] Lumpkin MM (1997). Reports of diabetes and hyperglycemia in patients receiving protease inhibitors for the treatment of human immunodeficiency virus (HIV), FDA Public Advisory.

[18] Dever LL, Oruwari PA, Figueroa WE, O’Donovan CA, Eng RH (2000). Hyperglycemia associated with protease inhibitors in an urban HIV-infected minority patient population. Ann Pharmacother.

[19] Tsiodras S, Mantzoros C, Hammer S, Samore M (2000). Effects of Protease inhibitors on hyperglycemia, hyperlipidemia and lipodystrophy. A 5-year cohort study. Arch Intern Med.

[20] Carr A, Samaras K, Thorisdottir A, Kaufmann GR, Chisholm DJ, Cooper DA (1999). Diagnosis, prediction, an natural course of HIV-1 protease-inhibitor-associated lipodystrophy, hyperlipidemia, and diabetes mellitus: a cohort study. Lancet.

[21] Saves M, Francois R, Jacqueline C, Rozenbaum W, Ragnaud J-M, Perronne C (2002). Factors related to lipodystrophy and metabolic alterations in patients with Human immunodeficiency virus infection receiving highly active antiretroviral therapy. Clin Infec Dis.

[22] Koster JC, Remedi MS, Qiu H, Nichols CG, Hruz PW (2003). HIV protease inhibitors acutely impair glucose-stimulated insulin release. Diabetes.

[23] Dufer M, Neye Y, Krippeit-Drews P, Drews G. Direct interference of HIV protease inhibitors with pancreatic beta-cell function. Naunyn Schmiedebergs *Arch Pharmacol*. 2004;369:583–90. 10.1007/s00210-004-0933-6 15197535

[24] Martinez E, Conget I, Loranzo L, Casamitjana R, Gatell JM (1999). Reversion of metabolic abnormalities after switching from HIV-1 protease inhibitors to nevirapine. AIDS.

[25] Woerle HJ, Mariuz PR, Meyer C, Reichman RC, Popa EM, Dostou JM (2003). Mechanisms for the deterioration in glucose tolerance associated with HIV protease inhibitor regimens. Diabetes.

[26] Ige OO, Yilgwan CS, Ebonyi AO, Adah R, Adedeji I, Yiltok ES (2017). Serum lipid and glucose profiles in HIV-positive Nigerian children. J Virus Erad.

[27] Chantry CJ, Hughes MD, Alvero C, Cervia JS, Meyer WA 3. Lipid and glucose alterations in HIV-infected children beginning or changing antiretroviral therapy. Pediatrics. 2008;122:e129-38. rd et al. 10.1542/peds.2007-2467 PMC2782494 18519448

[28] Vigano A, Brambilla P, Pattarino G, Stucchi S, Fasan S, Raimondi C (2009). Long-term evaluation of glucose homeostatsis in a cohort of HAART-treated HIV-infected children. A longitudinal, observational cohort study. Clin Drug Invest.

[29] Dejkhamron P, Unachak K, Aurpibul L, Sirisanthana V (2014). Insulin resistance and lipid profiles in HIV-infected Thai children receiving lopinaviréritonavir-based highly active antiretroviral therapy. J Pediatr Endocrinol metab.

[30] Ngondi J, Mbouobda HD, Fonkoua M, Nouemsi APK, Oben J (2007). The long-term effect of different combination therapies on glucose metabolism in HIV/AIDS subjects in Cameroon. J Med Sci.

[31] Brown TT, Tassiopoulos K, Bosch RJ, Shikuma C, McComsey GA (2010). Association between systemic inflammation and incident diabetes in HIV-infected patients after initiation of antiretroviral therapy. Diabetes Care.

